# A snapshot of *Plasmodium falciparum* malaria drug resistance markers in Sudan: a pilot study

**DOI:** 10.1186/s13104-020-05363-0

**Published:** 2020-11-07

**Authors:** Nouh S. Mohamed, Hanadi Abdelbagi, Hussam A. Osman, Abdallah E. Ahmed, Alaa M. Yousif, Yusraa B. Edris, Eman Y. Osman, Aahd R. Elsadig, Emmanuel E. Siddig, Madinna Mustafa, Ammar A. Mohammed, Yousif Ali, Maha M. Osman, Mohamed S. Ali, Rihab A. Omer, Ayman Ahmed, Carol H. Sibley

**Affiliations:** 1 Department of Molecular Parasitology and Medical Entomology, Faculty of Medical Laboratory Sciences, Nile University, Khartoum, Sudan; 2 Department of Biotechnology, School of Pharmacy, Ahfad University for Women, Omdurman, Sudan; 3 Alfarrabi College for Science and Technology, Khartoum, Sudan; 4 Parasitology and Medical Entomology Department, Faculty of Medicine, Sinnar University, Sinnar, Sudan; 5 Alraqi Hospital, National University, Khartoum, Sudan; 6 Mycetoma Research Center, University of Khartoum, Khartoum, Sudan; 7 Sudan Federal Ministry of Health, Khartoum, Sudan; 8 Faculty of Medicine, Neelain University, Khartoum, Sudan; 9 Department of Molecular Biology, Institute of Parasitology, University of Leipzig, Leipzig, Germany; 10 Institute of Endemic Diseases, University of Khartoum, Khartoum, Sudan; 11 University of Washington, Seattle, USA

**Keywords:** *Plasmodium falciparum*, Molecular markers, Multi drug resistance, Sudan

## Abstract

**Objectives:**

Malaria infection is still known to be a worldwide public health problem, especially in tropical and sub-tropical African countries like Sudan. A pilot study conducted to describe the trend of *P. falciparum* drug resistance markers in 2017–2018 in comparison to CQ and AS/SP eras in Sudan. The *Pfcrt*, *Pfmdr-1*, *Pfdhfr*, and *Pfdhps* genes were investigated. Data deposited by the worldwide antimalarial resistance network was consulted, and the molecular markers previously reported from Sudan were analyzed.

**Results:**

Drug molecular markers analysis was successfully done on 20 *P. falciparum* isolates. The *Pfcrt* K76 showed high frequency; 16 (80%). For the *Pfmdr-1,* 9 (45%) isolates were carrying the N86 allele, and 11 (55%) were 86Y allele. While the Y184F of the *Pfmdr-1* showed a higher frequency of 184F compared to Y184; 16 (80%) and 4 (20%), respectively. In the *Pfdhfr*, 51I allele showed higher frequency compared to N51; 18 (90%) and 2 (10%), respectively. For S108N, 18 (90%) were 108 N and 2 (10%) were S108. In the *Pfdhps*, all isolates were carrying the mutant alleles; 437G and 540E. The frequency distribution of the *Pfcrt*, *Pfmdr-1*, *Pfdhfr*, *Pfdhps* was significantly different across the whole years in Sudan.

## Introduction

Malaria infection is still known to be a worldwide public health problem, especially in tropical and sub-tropical African countries [1]. In Sudan, in the late 70s of the last century, a high proportion of drug resistance was reported when Chloroquine (CQ) was introduced as the first-line treatment for falciparum malaria [2]. By 2004 the malaria treatment protocol was shifted to the artemisinin-based combination treatment (ACTs); Artesunate (AS) and Sulfadoxine–Pyrimethamine (SP) as a first-line treatment against uncomplicated falciparum malaria, and artemether–lumefantrine (AL) as a second-line treatment [3]. In 2017, the malaria treatment protocol was shifted to AL for the treatment of uncomplicated falciparum malaria, and Quinine for treating the severe infections [4]. Subsequently, therapeutic efficacy studies were conducted to monitor and detect the emergence of drug-resistant malaria parasites [5–9]. However, another approach for the early detection of drug resistance emergence was implemented by using molecular markers to investigate the efficacy of treatments in-vitro.

*P. falciparum* chloroquine resistance transporter (*Pfcrt*) and *P. falciparum* multidrug resistance gene 1 (*Pfmdr-1*) are previously known membrane transporters associated with resistance to the drug combination of CQ and AQ or MQ and Lumefantrine (L) [10, 11]. The CVIET haplotype of *Pfcrt* is known as the most robust CQ resistance marker in Africa [12–15]. In vitro experiments showed that N86Y and Y184F mutations in the *Pfmdr-1* gene increases the inhibitory concentrations of CQ and AQ [10, 12], and reduce susceptibility to MQ and L [12, 13].

Previous studies on the *P. falciparum* dihydrofolate reductase (*Pfdhfr*) and dihydropteroate synthase (*Pfdhps*) has been identified as known targets of SP antimalarial drugs [14]. The mutations in the *Pfdhfr* codons 51, 59, and 164; and the *Pfdhps* codons 436, 437, 540, 581, and 613 were conferring the resistance [14].

The need for updated molecular markers studies to investigate the frequency of falciparum malaria drug-resistant is extreme. This pilot study aims to describe the trend of *P. falciparum* drug resistance markers in 2017–2018 in comparison to CQ and AS/SP eras in Sudan.

## Materials and methods

This pilot study was conducted in Khartoum state between December 2017 and July 2018. Febrile patients (axillary temperature < 37 °C) who were diagnosed microscopically by examining Giemsa stained blood films as falciparum malaria infection were recruited. Informed consent was taken from the patients before sample collection. Participants diagnosed with *P. falciparum/P. vivax* co-infection and *P. vivax* mono-infections were excluded.

### Sample collection and DNA extraction

Two ml blood samples were collected before starting the treatment and preserved into lithium heparin blood containers for DNA extraction using the Guanidine Chloride extraction method as described previously [15]. DNA was stored in − 20 °C until molecular examinations later.

### Parasite genotyping and drug resistance markers assessment

The microscopic diagnosis was confirmed using the primers described previously [16]. Genotyping of the specific point mutations in the *P. falciparum* genome was done using the Sanger sequencing method by using the primers sets for *Pfcrt*, *Pfmdr-1*, *Pfdhfr*, and *Pfdhps* genes as described previously [17]. PCR amplicons were sequenced in both directions using the forward and reverse primers for each gene to exclude any base-calling errors that could be obtained during sequencing. Sequences were validated using GENtle software (v1.9.4) and aligned in comparison with the wildtype *P. falciparum* 3D7 strain reference sequences (PF3D7_0709000 for *Pfcrt*, PF3D7_0523000 for *Pfmdr-1*, PF3D7_1324800 for *Pfdhfr*, and PF3D7_0810800 for *Pfdhps*). The deduced amino acids were translated from nucleotide sequences using MEGA7 software (v7.0.26) to determine sequences mutations at the *Pfcrt* codon 76; *Pfmdr-1* codons 86 and 184; *Pfdhfr* codons 51, 59, and 108; and the substitutions at the *Pfdhps* in codons 437 and 540. The nucleotide sequences used in this study have been deposited in the NCBI GenBank database (https://www.ncbi.nlm.nih.gov/) under the accession numbers MT995200–MT995259.

### Previous reports on drug resistance markers in Sudan

Data deposited by the worldwide antimalarial resistance network (WWARN) (https://www.wwarn.org/) was consulted, and the molecular markers previously reported from Sudan were identified, collected, and analyzed to compare between past and present frequency of malaria drug resistance mutations. Data sets included SP molecular surveyors (https://www.wwarn.org/sp-molecular-surveyor) and ACT partner drug molecular surveyors (https://www.wwarn.org/tracking-resistance/act-partner-drug-molecular-surveyor). Numbers and drug molecular marker genotypes of *P. falciparum* isolates included in the historical literature review data set analyzed in this study are presented in Additional file 1.

### Statistical analysis

The statistical analysis was done using the statistical Package for Social Sciences (SPSS, v20.0). One-way ANOVA test was used to calculate the least significance difference of frequency distribution in the molecular markers. Pearson correlation was used to investigate the association between the different drug resistance markers. P value ≤ 0.05 was considered statistically significant.

## Results

### Molecular genotyping results

In this pilot study, a total of 28 malaria parasite isolates were genotyped, of them, 2 and 6 isolates were excluded since were *P. falciparum*/*P. vivax* coinfections and *P. vivax* infections, respectively. The remaining 20 isolates were confirmed by PCR as *P. falciparum* mono-infections.

### Frequency of *P. falciparum* drug resistance markers

Drug molecular markers analysis was successfully done on the 20 *P. falciparum* isolates. Out of the 20 isolates, *Pfcrt* K76 showed the highest frequency; 16 (80%). *Pfcrt* 76** T** was 4 (20%). None of the isolates was carrying mixed *Pfcrt* allele infection; K/**T**. For the *Pfmdr-1* marker, 9 (45%) isolates were carrying the N86 allele and 11 (55%) were carrying the 86**Y** allele. While the Y184**F** of the *Pfmdr-1* showed a higher frequency of 184**F** compared to Y184; 16 (80%) and 4 (20%), respectively. Concerning the double *Pfmdr-1* haplotypes; NY haplotype was 2 (10%), N**F** was 7 (35%), **YF** was 9 (45%), and **Y**Y was 2 (10%).

The *Pfdhfr* N51**I** showed a higher frequency of 51**I** compared to N51; 18 (90%) and 2 (10%), respectively. Whereas for Pfdhfr C59**R**, C59 was 18 (90%), and 59**R** was 2 (10%). For Pfdhfr S108**N**, 18 (90%) were 108** N** and 2 (10%) were S108. For the triplet haplotype of the *Pfdhfr*, the haplotype **I**C**N** was the most frequent; 16 (80%). **IRN** and NCS were only present in two isolates; 2 (10%) for each. For the *Pfdhps*, all the 20 (100%) isolates were carrying the mutant alleles; 437**G** and 540**E** (Table 1). A statistically significant positive correlation was observed for *Pfmdr-1* and the combined *Pfdhfr* and *Pfdhps* alleles, Pearson r’ = 0.509, P value = 0.035. While, for the *Pfcrt* and the combined *Pfdhfr* and *Pfdhps*, a statistically insignificant negative correlation was found, Pearson’s’ *r* = − 0.248, P value = 0.291.

**Table 1:** The distribution of multidrug resistance markers among the 2017–2018 study isolates

Isolate ID	*Pfcrt*	*Pfmdr-1*	*Pfdhfr*	*Pfdhps*
Isolate 1	K	**YF**	**I**C**N**	**GE**
Isolate 2	**T**	N**F**	**I**C**N**	**GE**
Isolate 3	K	**YF**	**I**C**N**	**GE**
Isolate 4	K	**Y**Y	**I**C**N**	**GE**
Isolate 5	**T**	N**F**	**I**C**N**	**GE**
Isolate 6	K	NY	**I**C**N**	**GE**
Isolate 7	K	**YF**	**IRN**	**GE**
Isolate 8	K	**YF**	**NCS**	**GE**
Isolate 9	K	NY	**I**C**N**	**GE**
Isolate 10	**T**	N**F**	**I**C**N**	**GE**
Isolate 11	K	N**F**	**I**C**N**	**GE**
Isolate 12	K	**YF**	NCS	**GE**
Isolate 13	K	N**F**	**I**C**N**	**GE**
Isolate 14	K	**YF**	**IRN**	**GE**
Isolate 15	**T**	N**F**	**I**C**N**	**GE**
Isolate 16	K	**YF**	**I**C**N**	**GE**
Isolate 17	K	**YF**	**I**C**N**	**GE**
Isolate 18	K	**Y**Y	**I**C**N**	**GE**
Isolate 19	K	**YF**	**I**C**N**	**GE**
Isolate 20	K	N**F**	**I**C**N**	**GE**

^*^Letters denotes the wildtype and mutant alleles of the *Pfcrt* K76T; *Pfmdr-1* N86Y and Y184F; *Pfdhfr* N51I, C59R, and S108N; *Pfdhps* A437G and K540E. Mutant alleles were written in bold

### The trend in *P. falciparum* multidrug resistance from 1989 to 2018

**Fig. 1:**
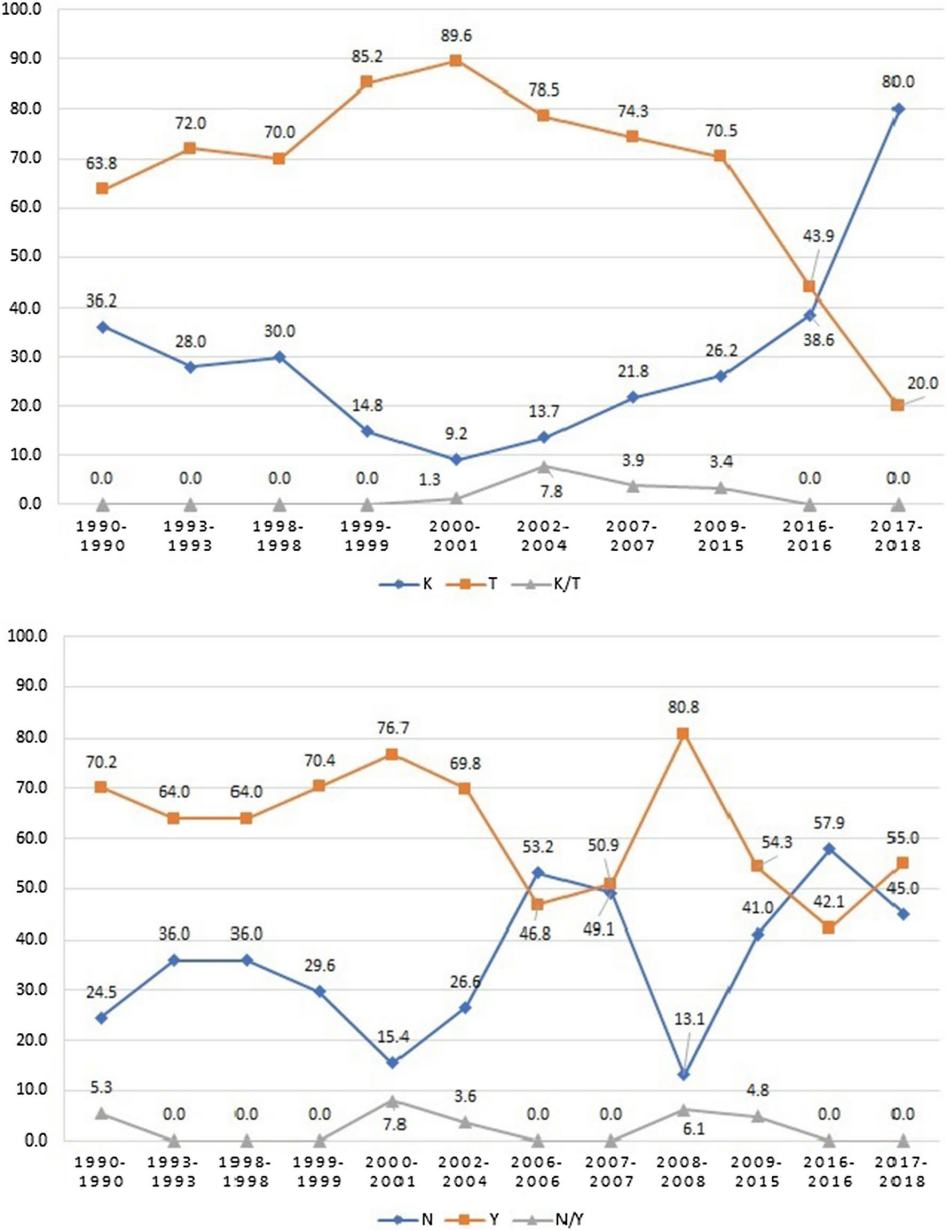
Frequency distribution of single *Pfcrt* K76**T** and *Pfmdr-1* N86**Y** genotypes in 2017–2018 samples compared with previously published reports

The **T** allele of the *Pfcrt* was at a higher frequency during 2000–2001 (89.6%) however, **T** mutant allele frequency started to dropdown reaching up to 43.9% in 2016 and bottomed at 20% in 2018. Frequency of *Pfcrt* K76 allele was higher compared to all previous years; 80%, while the N86**Y** mutation of the *Pfmdr-1* was extremely flocculation during the past years (Fig. 1).

**Fig. 2:**
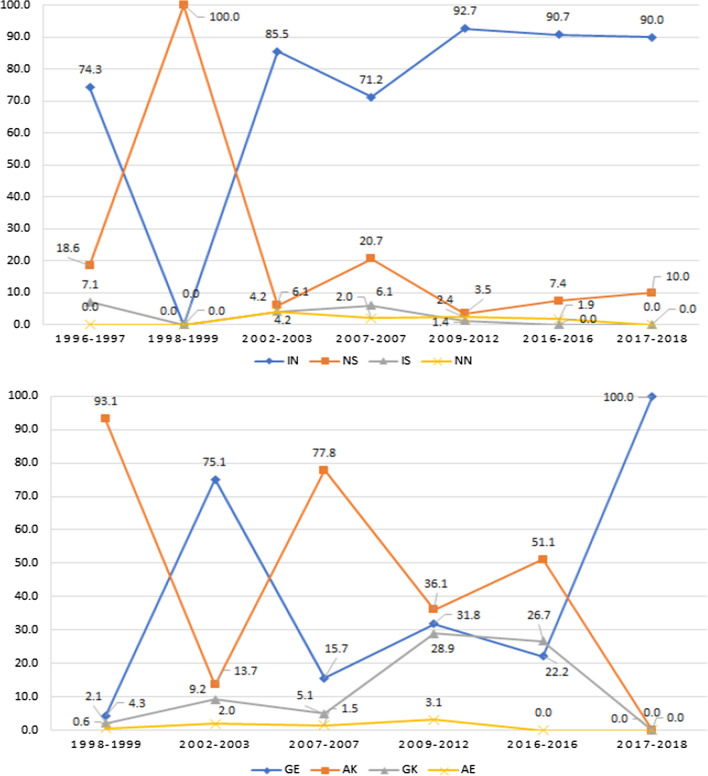
Frequency distribution of double haplotypes of *Pfdhfr* N51**I** and S108**N**, and *Pfdhps* A437**G** and K540**E** in 2017–2018 samples compared with previously published reports

Concerning the *Pfdhfr* N51**I** and S108**N,** in 1996–1997 the NS wildtype haplotype showed low frequency compared to the **IN** mutant haplotype; 18.6% and 74.3%, respectively. While in 1998–1999 the frequency of the NS haplotype reached to 100%. Whereas the prevalence of the NS haplotype from 2002–2003 continued to decrease to 10% in 2017–2018. On the other hand, the **IN** mutant haplotype increased to 85.5% in 2002–2003, and reaching 92.7% in 2009–2012; and remained constant approximately 90% in 2017–2018. Also, for the *Pfdhps*, in 1998–1999 AK wildtype haplotype was 93.1%, but in 2002–2003 **GE** mutant haplotype increased to 75.1%. In 2007, the AK wildtype haplotype increased again to 77.8% and decreased to 36.1% in 2009–2012. And, in 2016 reached 51.1%. However, in this study in 2017–2018, the **GE** mutant haplotype was prevalent in all the studied samples 20 (100%) (Fig. 2).

The frequency distribution of the *Pfcrt* and *Pfmdr-1*, *Pfdhfr*, and *Pfdhps* mutations was significantly different across the whole years in Sudan. An illustrated statistical significance and insignificance of the frequency distribution of *P. falciparum* multidrug resistance markers between the different years' intervals are described in Additional file 2 Tables S1–S5.

## Discussion

The reported frequency of *Pfcrt* K76 allele in this study was significantly high compared to previous years, especially during the CQ deployment years in Sudan [9]. During the AS/SP era; since 2004, *Pfcrt* K76 allele had increased. This increase can be suggested due to limited or ambient exposure of *P. falciparum* parasite to CQ or might be due to an increase in AL pressure. Similar results were reported from Malawi, and Tanzania supporting the increase of *Pfcrt* K76 allele [18–21]. While, in Zambia, complete disappearance of *Pfcrt* 76 T allele from the examined isolates was reported [22].

The *Pfdhfr* **IN** haplotype and *Pfdhps* **GE** haplotype significantly increased in 2017–2018 where both haplotypes were showing high frequency; 90% and 100%, respectively. This increase could be due to the enduring pressure of AS/SP since 2007 [23]. This phenomenon was observed in all countries where AS/SP was used to treat uncomplicated malaria such as Nigeria and Equatorial Guinea; where the prevalence of mutant haplotypes reached more than 90% [24–26].

The prevalence of *Pfmdr-1* N**F** haplotype in 2017–2018 is noted among 35% of the study samples. Comparison between previous years for the frequency distribution of *Pfmdr-1* double haplotypes showed a statistically significant difference between all years and 2008 when at that time the recommended malaria treatment was AS/SP, and since 2009 the use of AL is significantly increased due to malpractice in drug use, such as usage of incorrect dosage and insufficient information stated to patients about the prescribed treatment which may lead to the increase in resistance and recurrent infections rates [27].

Previously, the development of molecular markers for AL resistance was thought to be difficult because there were no known resistant lab lines can be used as resistance controls. Meanwhile, investigating the *Pfmdr-1* N**FSND** haplotype could be the role evolution for the developed mutations because it allows a longer survival rate of the parasite [11, 28].

In the present study, *Pfmdr-1* 86Y allele, *Pfdhfr* **IN** haplotype, and *Pfdhps* **GE** haplotype were constituting the majority of the studied samples; 90%, which is also similar to a previous study conducted in Sudan; where all the investigated parasite isolates were carrying the *Pfmdr-1* 86**Y** allele, *Pfdhfr* **IN** haplotype, and *Pfdhps* 540**E** [23].

The prevalence of *Pfdhps* double haplotype **GE** detected in this study could hinder the effect of SP if used as intermittent preventive therapy during pregnancy (IPTp). Although, SP as IPTp was not implemented and there is no information about the use of SP during pregnancy in Sudan [29]. The presence of *Pfdhfr* **IRN** in combination with *Pfdhps* **GE** haplotypes forming the quintuple mutant haplotype confers a high risk for treatment failure in malaria-infected children and nonpregnant adults who receive SP as a seasonal malaria chemoprevention treatment (SMC-SP) [30]. However, previous studies indicated that IPTp-SP is still efficacious in areas with a high prevalence of resistant *P. falciparum* parasite [31]. Nevertheless, the increased resistance rate might compromise the implication of IPTp-SP [32–34].

## Conclusion

This study describes the distribution of *P. falciparum* multidrug resistance markers throughout Sudan. The study provides a baseline data of the status of these markers which could be very useful for the malaria control program for establishing surveillance system to monitor the emergence of malaria drug resistance for more effective treatment protocol and successful control of the disease.

### Limitations

- The lack of clinical and background information, particularly, the previously used drug before the blood sampling substantially can affect the prevalence of the alleles. Therefore, complete clinical history information is needed and can be very useful in future studies.

## Supplementary information

**Additional file 1: Table S1.** Numbers and drug molecular markers genotypes of *P. falciparum* isolates from Sudan 1989 – 2018.

**Additional file 2: Table S1.** Statistical significance of Pfcrt K76T frequency distribution across the different previous studies conducted in Sudan. **Table S2.** Statistical significance of Pfmdr-1 N86Y frequency distribution across the different previous studies conducted in Sudan. **Table S3.** Statistical significance of Pfdhfr N51I and S108N double haplotype frequency distribution across the different previous studies conducted in Sudan. **Table S4.** Statistical significance of Pfdhps A437G and K540E double haplotype frequency distribution across the different previous studies conducted in Sudan. **Table S5.** Statistical significance of Pfmdr-1 N86Y and Y184F double haplotype frequency distribution across the different previous studies conducted in Sudan.

## Data Availability

All datasets used and analysed in this study are available in the manuscript. DNA sequences analysed during the current study are available at the NCBI GenBank database under the accession numbers MT995200–MT995259.

## Abbreviations

ACTs: Artemisinin-based combination therapies
AL: Artemether–Lumefantrine
AQ: Amodiaquine-hydrochloride
AS: Artesunate
CQ: Chloroquine
IPTp: Intermittent preventive therapy during pregnancy
L: Lumefantrine
MQ: Mefloquine
*Pfcrt*: *P. falciparum* Chloroquine resistance transporter
*Pfdhfr*: *P. falciparum* Dihydrofolate reductase
*Pfdhps*: *P. falciparum* Dihydropteroate synthase
*Pfmdr-1*: *P. falciparum* Multidrug resistance gene 1
SP: Sulfadoxine–Pyrimethamine
WWARN: Worldwide Antimalarial Resistance Network
