# Supplementary material for: A snapshot of Plasmodium falciparum malaria drug resistance markers in Sudan: a pilot study
Source: BMC Res Notes. 2020 Nov 7;13:512. doi: 10.1186/s13104-020-05363-0 (PMC7648977; doi:10.1186/s13104-020-05363-0)
Supplement: Supplementary file 1 — Additional file 1: Table S1. Numbers and drug molecular markers genotypes of P. falciparum isolates from Sudan 1989 – 2018. [file 13104_2020_5363_MOESM1_ESM.docx]

**Additional file 1:**

**Table 1: Numbers and drug molecular markers genotypes of *P. falciparum* isolates from Sudan 1989 – 2018.**

|  | K | T | Total | N | Y | Total | IN | NS | IS | NN | Total | GE | AE | GK | AK | Total |
| --- | --- | --- | --- | --- | --- | --- | --- | --- | --- | --- | --- | --- | --- | --- | --- | --- |
| Gedaref | | | | | | | | | | | | | | | | |
| 1989-1990 | 34 | 60 | 94 | 23 | 66 | 89 | 0 | 218 | 0 | 0 | 218 | 0 | 0 | 0 | 44 | 44 |
| 1993-1993 | 14 | 36 | 50 | 18 | 32 | 50 | - | - | - | - | - | 0 | 0 | 0 | 50 | 50 |
| 1998-1999 | 27 | 104 | 131 | 42 | 89 | 131 | - | - | - | - | - | 35 | 1 | 12 | 342 | 390 |
| 2000-2001 | 35 | 274 | 309 | 53 | 264 | 317 | - | - | - | - | - | 31 | 1 | 0 | 54 | 86 |
| 2002-2004 | 16 | 190 | 206 | 81 | 213 | 294 | 316 | 38 | 10 | 10 | 374 | 110 | 47 | 25 | 20 | 202 |
| 2006-2006 | - | - | - | 25 | 22 | 47 | - | - | - | - | - | - | - | - | - | - |
| 2007-2007 | 11 | 91 | 102 | 88 | 110 | 198 | 21 | 3 | 0 | 3 | 27 | 6 | 0 | 2 | 19 | 27 |
| 2008-2008 | - | - | - | 13 | 80 | 93 | - | - | - | - | - | - | - | - | - | - |
| Khartoum | | | | | | | | | | | | | | | | |
| 1996-1997 | - | - | - | - | - | - | 52 | 13 | 5 | 0 | 70 | - | - | - | - | - |
| 1998-1999 | - | - | - | - | - | - | - | - | - | - | - | 6 | 0 | 0 | 145 | 151 |
| 2001-2001 | 0 | 78 | 78 | 6 | 67 | 73 | - | - | - | - | - | - | - | - | - | - |
| 2012-2015 | 14 | 26 | 40 | 22 | 9 | 31 | - | - | - | - | - | - | - | - | - | - |
| 2016-2016 | 26 | 41 | 67 | 33 | 24 | 57 | 49 | 4 | 0 | 1 | 54 | 10 | 0 | 12 | 23 | 45 |
| 2017-2018 | 16 | 4 | 20 | 9 | 11 | 20 | 18 | 0 | 0 | 2 | 20 | 20 | 0 | 0 | 0 | 20 |
| Gezira | | | | | | | | | | | | | | | | |
| 2007-2007 | 51 | 120 | 171 | 84 | 87 | 171 | 116 | 38 | 12 | 5 | 171 | 25 | 3 | 8 | 135 | 171 |
| 2009-2012 | 13 | 63 | 76 | 27 | 47 | 74 | 69 | 4 | 0 | 2 | 75 | 2 | 14 | 46 | 13 | 75 |
| Upper Nile | | | | | | | | | | | | | | | | |
| 2002-2002 | 33 | 56 | 89 | 61 | 28 | 89 | - | - | - | - | - | - | - | - | - | - |
| 2009-2012 | 23 | 45 | 68 | 39 | 35 | 74 | 66 | 1 | 3 | 3 | 73 | 0 | 1 | 37 | 35 | 74 |
| South Darfur | | | | | | | | | | | | | | | | |
| 2009-2012 | - | - | - | 22 | 52 | 74 | 62 | 1 | 0 | 11 | 74 | 0 | 1 | 53 | 15 | 69 |
| South Kordofan | | | | | | | | | | | | | | | | |
| 2003-2004 | - | - | - | 13 | 143 | 156 | 11 | 28 | 0 | 121 | 160 | - | - | - | - | - |
| Kassala | | | | | | | | | | | | | | | | |
| 2009-2012 | - | - | - | 83 | 62 | 145 | 168 | 2 | 4 | 0 | 174 | 0 | 0 | 48 | 36 | 84 |

Letters denotes the wildtype and mutant alleles of the *Pfcrt* K76T; *Pfmdr1* N86Y and Y184F; *Pfdhfr* N51I and S108N; *Pfdhps* A437G and K540E. Where no drug molecular marker study been conducted is represented by ( - ).
