# Supplementary material for: A snapshot of Plasmodium falciparum malaria drug resistance markers in Sudan: a pilot study
Source: BMC Res Notes. 2020 Nov 7;13:512. doi: 10.1186/s13104-020-05363-0 (PMC7648977; doi:10.1186/s13104-020-05363-0)
Supplement: Supplementary file 2 — Additional file 2: Table S1. Statistical significance of Pfcrt K76T frequency distribution across the different previous studies conducted in Sudan. Table S2. Statistical significance of Pfmdr-1 N86Y frequency distribution across the different previous studies conducted in Sudan. Table S3. Statistical significance of Pfdhfr N51I and S108N double haplotype frequency distribution across the different previous studies conducted in Sudan. Table S4. Statistical significance of Pfdhps A437G and K540E double haplotype frequency distribution across the different previous studies conducted in Sudan. Table S5. Statistical significance of Pfmdr-1 N86Y and Y184F double haplotype frequency distribution across the different previous studies conducted in Sudan. [file 13104_2020_5363_MOESM2_ESM.docx]

**Supplementary file 2:**

**Table S1:** statistical significance of *Pfcrt* K76T frequency distribution across the different previous studies conducted in Sudan

| K76T | 1989-1990 | 1993-1993 | 1998-1998 | 1999-1999 | 2000-2001 | 2002-2003 | 2007-2007 | 2009-2015 | 2016-2016 |
| --- | --- | --- | --- | --- | --- | --- | --- | --- | --- |
| 1989-1990 | - | - | - | - | - | - | - | - | - |
| 1993-1993 | 0.249 | - | - | - | - | - | - | - | - |
| 1998-1998 | 0.384 | 0.805 | - | - | - | - | - | - | - |
| 1999-1999 | 0.001 | 0.071 | 0.037 | - | - | - | - | - | - |
| 2000-2001 | 0.000 | 0.002 | 0.001 | 0.244 | - | - | - | - | - |
| 2002-2003 | 0.000 | 0.030 | 0.012 | 0.996 | 0.040 | - | - | - | - |
| 2007-2007 | 0.006 | 0.396 | 0.242 | 0.124 | 0.000 | 0.012 | - | - | - |
| 2009-2015 | 0.000 | 0.000 | 0.000 | 0.000 | 0.000 | 0.000 | 0.000 | - | - |
| 2016-2016 | 0.684 | 0.154 | 0.245 | 0.000 | 0.000 | 0.000 | 0.004 | 0.001 | - |
| 2017-2018 | 0.376 | 0.113 | 0.162 | 0.003 | 0.000 | 0.001 | 0.018 | 0.189 | 0.549 |

**Table S2:** statistical significance of *Pfmdr-1* N86Y frequency distribution across the different previous studies conducted in Sudan

| N86Y | 1989-1990 | 1993-1993 | 1998-1998 | 1999-1999 | 2000-2001 | 2002-2004 | 2006-2006 | 2007-2007 | 2008-2008 | 2009-2015 | 2016-2016 |
| --- | --- | --- | --- | --- | --- | --- | --- | --- | --- | --- | --- |
| 1989-1990 | - | - | - | - | - | - | - | - | - | - | - |
| 1993-1993 | 0.058 | - | - | - | - | - | - | - | - | - | - |
| 1998-1998 | 0.058 | 1.000 | - | - | - | - | - | - | - | - | - |
| 1999-1999 | 0.173 | 0.485 | 0.485 | - | - | - | - | - | - | - | - |
| 2000-2001 | 0.050 | 0.000 | 0.000 | 0.000 | - | - | - | - | - | - | - |
| 2002-2004 | 0.525 | 0.092 | 0.092 | 0.292 | 0.000 | - | - | - | - | - | - |
| 2006-2006 | 0.000 | 0.096 | 0.096 | 0.011 | 0.000 | 0.000 | - | - | - | - | - |
| 2007-2007 | 0.000 | 0.108 | 0.108 | 0.004 | 0.000 | 0.000 | 0.626 | - | - | - | - |
| 2008-2008 | 0.099 | 0.001 | 0.001 | 0.003 | 0.933 | 0.007 | 0.000 | 0.000 | - | - | - |
| 2009-2015 | 0.004 | 0.982 | 0.982 | 0.293 | 0.000 | 0.001 | 0.030 | 0.006 | 0.000 | - | - |
| 2016-2016 | 0.000 | 0.026 | 0.026 | 0.001 | 0.000 | 0.000 | 0.638 | 0.258 | 0.000 | 0.003 | - |
| 2017-2018 | 0.039 | 0.503 | 0.503 | 0.225 | 0.001 | 0.060 | 0.545 | 0.731 | 0.002 | 0.448 | 0.328 |

**Table S3:** statistical significance of *Pfdhfr* N51I and S108N double haplotype frequency distribution across the different previous studies conducted in Sudan

| Pfdhfr | 1996-1997 | 1998-1999 | 2002-2003 | 2007-2007 | 2009-2012 | 2016-2016 |
| --- | --- | --- | --- | --- | --- | --- |
| 1996-1997 | - | - | - | - | - | - |
| 1998-1999 | 0.077 | - | - | - | - | - |
| 2002-2003 | 0.076 | 0.051 | - | - | - | - |
| 2007-2007 | 0.078 | 0.055 | 0.053 | - | - | - |
| 2009-2012 | 0.071 | 0.044 | 0.041 | 0.045 | - | - |
| 2016-2016 | 0.102 | 0.085 | 0.084 | 0.086 | 0.079 | - |
| 2017-2018 | 0.142 | 0.131 | 0.13 | 0.132 | 0.127 | 0.147 |

**Table S4:** statistical significance of *Pfdhps* A437G and K540E double haplotype frequency distribution across the different previous studies conducted in Sudan

| Pfdhps | 1998-1999 | 2002-2003 | 2007-2007 | 2009-2012 | 2016-2016 |
| --- | --- | --- | --- | --- | --- |
| 1998-1999 | - | - | - | - | - |
| 2002-2003 | 0.000 | - | - | - | - |
| 2007-2007 | 0.239 | 0.000 | - | - | - |
| 2009-2012 | 0.232 | 0.000 | 0.039 | - | - |
| 2016-2016 | 0.587 | 0.000 | 0.270 | 0.922 | - |
| 2017-2018 | 0.000 | 0.013 | 0.000 | 0.000 | 0.000 |

**Table S5:** statistical significance of *Pfmdr-1* N86Y and Y184F double haplotype frequency distribution across the different previous studies conducted in Sudan

|  | 2008-2008 | 2009-2012 | 2016-2016 |
| --- | --- | --- | --- |
| 2008-2008 | - | - | - |
| 2009-2012 | 0.000 | - | - |
| 2016-2016 | 0.000 | 0.238 | - |
| 2017-2018 | 0.005 | 0.794 | 0.666 |
